# Host factors that modify *Plasmodium falciparum* adhesion to endothelial receptors

**DOI:** 10.1038/s41598-017-14351-7

**Published:** 2017-10-24

**Authors:** Almahamoudou Mahamar, Oumar Attaher, Bruce Swihart, Amadou Barry, Bacary S. Diarra, Moussa B. Kanoute, Kadidia B. Cisse, Adama B. Dembele, Sekouba Keita, Benoît Gamain, Santara Gaoussou, Djibrilla Issiaka, Alassane Dicko, Patrick E. Duffy, Michal Fried

**Affiliations:** 1 Malaria Research & Training Center, Faculty of Medicine, Pharmacy and Dentistry, University of Sciences Techniques and Technologies of Bamako, P.O. Box 1805, Bamako, Mali; 2 Biostatistics Research Branch, National Institute of Allergy and Infectious Diseases, NIH, Rockville, Maryland USA; 3 Université Sorbonne Paris Cité, Université Paris Diderot, Inserm, INTS, Unité Biologie Intégrée du Globule Rouge, Laboratoire d’Excellence GR-Ex, Paris, France; 4 Laboratory of Malaria Immunology and Vaccinology, National Institute of Allergy and Infectious Diseases, NIH, Rockville, Maryland USA

## Abstract

*P. falciparum* virulence is related to adhesion and sequestration of infected erythrocytes (IE) in deep vascular beds, but the endothelial receptors involved in severe malaria remain unclear. In the largest ever study of clinical isolates, we surveyed adhesion of freshly collected IE from children under 5 years of age in Mali to identify novel vascular receptors, and examined the effects of host age, hemoglobin type, blood group and severe malaria on levels of IE adhesion to a panel of endothelial receptors. Several novel molecules, including integrin α3β1, VE-cadherin, ICAM-2, junctional adhesion molecule-B (JAM-B), laminin, and cellular fibronectin, supported binding of IE from children. Severe malaria was not significantly associated with levels of IE adhesion to any of the 19 receptors. Hemoglobin AC, which reduces severe malaria risk, reduced IE binding to the receptors CD36 and integrin α5β1, while hemoglobin AS did not modify IE adhesion to any receptors. Blood groups A, AB and B significantly reduced IE binding to ICAM-1. Severe malaria risk varies with age, but age significantly impacted the level of IE binding to only a few receptors: IE binding to JAM-B decreased with age, while binding to CD36 and integrin α5β1 significantly increased with age.

## Introduction

Adhesion and sequestration of *P. falciparum*-infected erythrocytes (IE) underlie cerebral malaria^1^ and maternal malaria^2^. Sequestration confers a survival advantage on the parasite: mature stage IE accumulate in post-capillary venules and placental intervillous spaces, where they avoid immunologic surveillance in the spleen, and where low oxygen tension favors the growth of *P. falciparum*. *In vitro* studies have demonstrated that IE can bind to several endothelial surface molecules, including thrombospondin (TSP), CD36, intercellular adhesion molecule-1 (ICAM-1), vascular cell adhesion molecule-1 (VCAM-1), E-selectin, P-selectin, chondroitin sulfate A (CSA), PECAM-1/CD31, gC1qR/HABP1/p32, endothelial protein C receptor (EPCR), integrin α5β1, and possibly the integrin molecule α_V_β_3_^2–15^. Parasite adhesion to the endothelium is mediated by a family of proteins expressed on the surface of infected erythrocytes (IE) named *Plasmodium falciparum* erythrocyte membrane protein 1 (PfEMP1)^16^.

An understanding of the binding receptors involved in severe malaria syndromes will guide the development of anti-adhesion therapies and vaccines. Naturally occurring red cell variants, such as hemoglobins C and S, modify PfEMP1 expression as well as parasite adhesion to endothelial cells, suggesting a possible mechanism for their protective effects^17^. Earlier studies that examined the relationship between specific binding interactions and severe malaria have yielded inconsistent results. Studies in Thailand, Kenya and Malawi failed to associate ICAM-1- binding parasites with severe malaria^18–21^, while studies in Kenya^22^ and Tanzania^15^ found a significant association. Recent studies have implicated novel receptors, such as endothelial protein C receptor (EPCR) related to severe malaria in Tanzania^15^, and the receptor gC1qR/HABP1/p32 related to seizures in Mozambique^23^. In Kenya and Tanzania, parasites collected from children with severe malaria were more likely to bind to multiple receptors than other parasites^24^, possibly confounding the effort to associate individual receptors with disease.

Age is a key risk factor for severe malaria: children in malaria endemic areas develop immunity to severe malaria more rapidly than immunity that reduces parasite burden^25^. Separately, host genetic factors like hemoglobin AS and AC reduce the risk of severe malaria. In this study we quantified the level of IE adhesion to a panel of endothelial receptors and analyzed the relationship to host risk factors.

## Results

### Study population

The analysis of IE binding included 2904 parasite isolates collected from 1089 children aged 1–60 months (mean (SD) 24.9 (15.4)). 59 of the 2904 parasite isolates were collected from 52 children presenting with severe malaria. Severe malaria presentations included severe malaria anemia (n = 26), prostration (n = 23), repeated convulsions (n = 9), and coma (n = 1). There were no differences in age between children with severe and non-severe malaria. Severe malaria incidence rate analysis of the whole cohort showed that the rate of severe malaria increased in children aged >12 months (Table 1). Compared to children aged >12–24 months, relative risk in children aged 0–12 months was lower (estimated as 0.39, p = 0.004), while the relative risks among children in the other age strata were similar.

**Table 1:** Severe malaria incidence rates and relative risk by age.

Age (months)	Severe malaria events	Person-year	Rate	RR (95% CI)	P value
0–12	17	1751	0.00971	0.389 (0.204–0.742)	0.004
>12–24	30	1262	0.02377	Reference	
>24–36	21	731	0.02873	1.256 (0.696–2.266)	0.4
>36–48	9	338	0.02663	1.236 (0.581–2.632)	0.5
>48–60	3	88	0.03409	1.919 (0.572–6.440)	0.3

### Novel adhesion receptors

To establish the IE binding phenotype, we surveyed 2904 freshly collected clinical parasite isolates. The panel of receptors under study included molecules shown to support parasite adhesion in previous studies, as well as the novel receptors cellular fibronectin, laminin, members of the integrin and junctional adhesion molecule (JAM) families, VE-cadherin, vitronectin and ICAM-2.

The selection of candidate novel receptors for the study was based on the following observations: laminin plasma levels are increased during acute malaria infection, suggesting vascular injury that may expose extracellular matrix molecules (ECM) like laminin and possibly other ECM like cellular fibronectin that can then mediate IE adhesion^26,27^. Further, it has been reported that laboratory parasite line FCR3 binds cellular fibronectin^28^. Other receptors were selected based on their known role in leukocyte adhesion like junctional adhesion molecules (JAM)^29^, ICAM-2, VE cadherin, integrin types β1 and β3 constitutively expressed in vascular endothelium including brain microvessels^30^.

Each individual receptor, including all novel receptors selected for study, supported the binding of a subset of clinical samples (Table 2). The largest proportion of clinical isolates adhered to the glycoprotein CD36 (IE count >0, 79.9%; IE count >20, 57.5%), followed by integrin αvβ3 (IE count >0, 54.6%; IE count >20, 29.8%).

**Table 2:** Proportion of clinical parasite isolates binding to various endothelial receptors.

Receptor	n	IE count >0 (%)	IE count >20 (%)	mean binding (range)
CD36	2904	79.9	57.5	136.0 (0–9997)
TSP	2216	35.4	11.0	11.5 (0–1160)
ICAM-1	2901	47.4	24.0	27.5 (0–2752)
E-selectin	2901	36.4	11.8	9.8 (0–841)
P-Selectin	2897	35.3	13.5	12.1 (0–1093)
PECAM-1	2480	33.1	12.0	8.6 (0–429)
VCAM-1	2898	17.6	2.3	2.4 (0–903)
CSA	2894	16.4	1.3	1.4 (0–562)
EPCR	723	21.0	1.9	2.1 (0–240)
Integrin α5β1	2862	36.0	14.6	11.4 (0–498)
Vitronectin	2499	21.6	2.5	2.0 (0–184)
C. fibronectin	2901	35.7	9.7	6.4 (0–665)
Laminin	2899	26.7	5.2	4.1 (0–376)
Integrin α3β1	2897	34.6	12.8	10.2 (0–1131)
Integrin αvβ3	2861	54.6	29.8	30.3 (0–1378)
JAM-A	2502	19.5	1.8	1.5 (0–97)
JAM-B	2439	38.3	16.0	15.4 (0–1344)
VE-cadherin	2709	37.9	14.7	14.0 (0–1164)
ICAM-2	2873	37.8	12.1	12.0 (0–1040)

**Figure 1:**
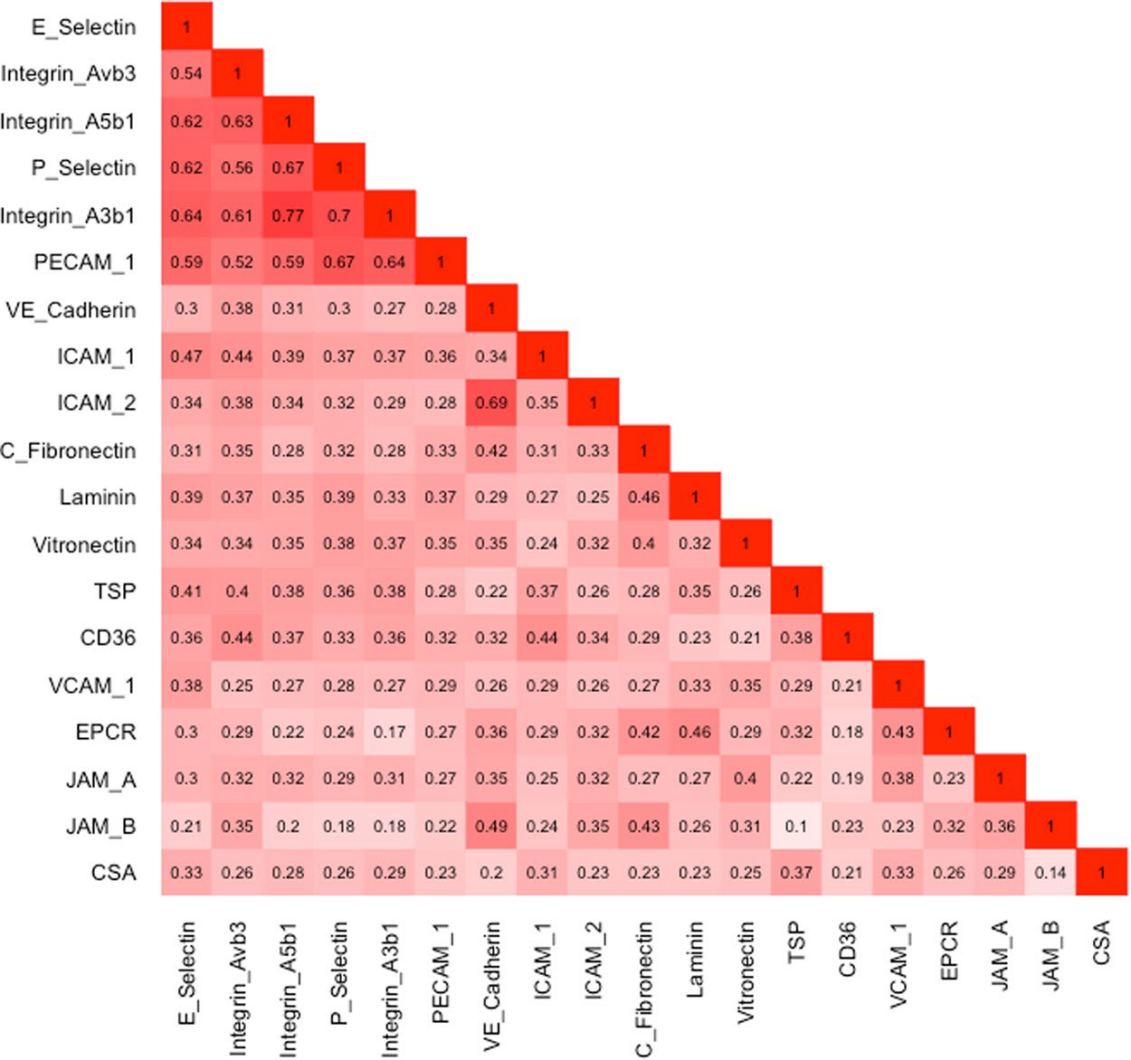
Correlation matrix of IE binding levels to endothelial receptors. Values within the box indicates the correlation value.

IE adhesion to the endothelium is mediated by members of the *Plasmodium falciparum* erythrocyte membrane protein 1 (PfEMP1) family. PfEMP1s have been grouped according to upstream sequences and further subgrouped based on domain types and domain combinations called “domain cassettes”^31^. We hypothesized that IE adhesion to different receptors will correlate if the adhesion interactions are mediated by different domains of the same PfEMP1. We therefore examined the correlations between IE binding levels to the various receptors, and found a large cluster of correlated interactions with the receptors integrin α3β1, integrin α5β1, integrin αvβ3, P-selectin, E-selectin and PECAM-1 (Fig. 1).

### Factors influencing parasite adhesion

After establishing the panel of potential binding receptors, we analyzed levels of IE adhesion for their relationships to host risk factors and to disease severity. Because parasite densities vary between assays, a B-spline regression model was applied (Table 3). This method allows a non-linear relationship to be flexibly modeled. However, the relationships between host factors, disease severity and levels of IE binding to the receptors panel were similar in linear regression models adjusted for parasite density with and without a B-spline.

**Table 3:** Factors associated with IE binding to endothelial receptors.

Receptor	HbAC^a^		HbAS^a^		Age^b^		Blood group A^c^		Blood group AB^c^		Blood group B^c^		Severe malaria^d^	
	Coefficient (SE)	P value	Coefficient (SE)	P value	Coefficient (SE)	P value	Coefficient (SE)	P value	Coefficient (SE)	P value	Coefficient (SE)	P value	Coefficient (SE)	P value
CD36	−86.49 (20.12)	<0.0001	−62.53 (26.76)	0.02	1.85 (0.42)	<0.0001	12.424 (15.73)	0.43	−13.41 (26.48)	0.61	13.40 (15.39)	0.38	−16.52 (43.14)	0.7
TSP	−0.96 (3.43)	0.78	−1.48 (4.56)	0.75	0.13 (0.07)	0.07	−5.34 (2.67)	0.046	−7.71 (4.50)	0.09	−4.81 (2.64)	0.07	3.70 (7.68)	0.6
ICAM-1	−15.23 (5.51)	0.006	0.65 (7.35)	0.93	0.30 (0.11)	0.01	−25.29 (4.31)	<0.0001	−28.18 (7.25)	<0.0001	−22.16 (4.22)	<0.0001	7.56 (11.81)	0.5
E-selectin	−6.83 (2.71)	0.012	−0.74 (3.61)	0.84	0.04 (0.06)	0.5	−3.65 (2.12)	0.09	−6.46 (3.58)	0.07	−5.65 (2.08)	0.007	3.69 (5.82)	0.5
P-Selectin	−8.50 (3.06)	0.006	−1.52 (4.06)	0.71	0.06 (0.06)	0.4	−5.07 (2.39)	0.03	−2.29 (4.03)	0.57	−6.09 (2.34)	0.009	0.97 (6.56)	0.8
PECAM-1	−6.23 (2.16)	0.004	−3.06 (2.78)	0.27	0.02 (0.05)	0.6	1.93 (1.66)	0.24	0.62 (2.69)	0.82	1.74 (1.62)	0.28	1.80 (4.41)	0.7
VCAM-1	−1.32 (1.53)	0.39	−1.60 (2.04)	0.43	0.05 (0.03)	0.09	−1.52 (1.20)	0.20	−1.28 (2.02)	0.53	−1.53 (1.17)	0.19	−2.34 (3.29)	0.5
CSA	−0.93 (0.78)	0.23	0.81 (1.03)	0.43	0.04 (0.02)	0.02	−0.99 (0.61)	0.10	−1.38 (1.02)	0.18	−1.08 (0.59)	0.07	0.78 (1.66)	0.6
EPCR	3.45 (1.51)	0.02	−0.69 (2.14)	0.75	0.008 (0.03)	0.80	−2.23 (1.24)	0.07	−0.74 (2.13)	0.73	−1.16 (1.14)	0.31	−2.72 (3.35)	0.4
Integrin α5β1	−8.37 (2.23)	<0.0001	−3.00 (2.98)	0.32	0.18 (0.05)	<0.0001	−2.73 (1.74)	0.12	−5.39 (2.92)	0.07	−4.67 (1.70)	0.006	1.77 (4.75)	0.7
Vitronectin	−0.90 (0.55)	0.10	−0.04 (0.73)	0.96	0.02 (0.01)	0.09	−0.10 (0.43)	0.80	−0.71 (0.71)	0.32	−0.46 (0.42)	0.28	−0.86 (1.21)	0.5
Cellular fibronectin	−0.68 (1.45)	0.64	−1.52 (1.92)	0.54	−0.007 (0.03)	0.82	−2.43 (1.13)	0.03	−2.60 (1.90)	0.17	−1.42 (1.11)	0.20	0.70 (3.10)	0.8
Laminin	1.00 (1.02)	0.34	0.20 (1.35)	0.88	−0.007 (0.02)	0.73	−1.45 (0.79)	0.07	−2.45 (1.34)	0.07	−1.10 (0.78)	0.15	2.10 (2.18)	0.3
Integrin α3β1	−7.37 (2.42)	0.003	−0.52 (3.21)	0.88	0.14 (0.05)	0.005	−3.17 (1.89)	0.09	−4.90 (3.18)	0.12	−3.95 (1.85)	0.03	3.14 (5.18)	0.5
Integrin αvβ3	−12.72 (4.86)	0.009	−1.24 (6.43)	0.85	0.18 (0.10)	0.07	−5.70 (3.79)	0.13	−6.16 (6.36)	0.33	−5.98 (3.71)	0.11	−3.16 (10.32)	0.8
JAM-A	−0.72 (0.41)	0.08	0.61 (0.54)	0.26	0.02 (0.008)	0.008	−0.27 (0.32)	0.39	0.015 (0.53)	0.98	−0.03 (0.31)	0.92	−0.27 (0.90)	0.8
JAM-B	−0.70 (4.06)	0.86	11.98 (5.64)	0.03	−0.41 (0.08)	<0.0001	−2.60 (3.18)	0.41	−4.03 (5.37)	0.45	1.61 (3.10)	0.60	−3.44 (8.60)	0.7
VE-cadherin	−5.70 (3.56)	0.11	5.06 (4.68)	0.28	0.10 (0.07)	0.15	−6.14 (2.76)	0.026	−4.60 (4.60)	0.32	0.81 (2.72)	0.77	−3.14 (7.79)	0.7
ICAM-2	−7.30 (2.98)	0.01	5.69 (3.94)	0.15	0.17 (0.06)	0.005	−3.39 (2.32)	0.15	−2.31 (3.90)	0.55	0.21 (2.27)	0.93	−2.19 (6.33)	0.7

P values of 0.0026 or less are considered significant. Models were adjusted for parasite density in the assay.

^a^HbAC and HbAS: Average change in binding level (IE count) compared to samples from children with HbAA.

^b^Age: Average change in binding level (IE count) for an increase of 1 month in age.

^c^Blood groups A, AB and B: Average change in binding level (IE count) compared to samples from children with blood group O.

^d^Severe malaria: Average change in binding level (IE count) compared to samples from children with non-severe malaria.

#### Hemoglobinopathies

The prevalence of HbAC (including 1 child with HbCC type) was 10.7% and HbAS (including 3 children with HbSS and 2 children with HbSC types) was 7.6%. Sickle cell trait did not significantly reduce IE adhesion to any of the receptors. Hemoglobin AC significantly reduced IE binding to the receptors CD36 and integrin α5β1, and its effect to reduce binding to integrin α3β1 approached significance (Table 3).

#### Blood group

Blood group O has been associated with reduced severe malaria risk in some but not all studies and has been associated with reduced rosetting (binding of uninfected to infected red cells)^32–39^. Here we examined whether ABO blood group modifies IE adhesion to endothelial receptors, and blood group O was used as a reference group (Table 3). Blood groups A, AB and B were associated with a significant reduction in IE adhesion to the receptor ICAM-1, while IE binding to other receptors was similar between blood group A, AB, B and O.

#### Age

Age affected the binding patterns to the various receptors in one of 3 ways: binding decreased as children aged; binding increased as children aged; binding was stable across the early years of life. IE binding to the receptor JAM-B significantly decreased with age. Conversely, IE binding to the receptors CD36 and integrin α5β1 significantly increased with age. IE binding to the receptors ICAM-1, ICAM-2 and integrin α3β1 also increased with age, and these relationships approached statistical significance. IE adhesion to the receptors TSP, E-selectin, PECAM-1, VCAM-1, P-selectin, CSA, EPCR, vitronectin, cellular fibronectin, laminin, integrin αvβ3, JAM-A and VE-cadherin did not change with age (Table 3).

#### Severe malaria

We examined the relationship between WHO-defined severe malaria and IE adhesion to specific endothelial receptors. The analysis included 59 parasite isolates collected from 52 children with severe malaria. The level of binding to all 19 receptors was similar between IE from severe and non-severe malaria cases (Table 3).

## Discussion

The pathological hallmark of *P. falciparum* parasites is their sequestration in deep vascular beds of various tissues. In the current study, we examined the effect of host factors that modify the risk of severe malaria on the level of IE binding to a panel of endothelial receptors. Age, hemoglobin AC, and blood group affect levels of IE adhesion to specific receptors.

We identified several novel receptors that support parasite adhesion, including laminin, cellular fibronectin, integrin α3β1, VE-cadherin, JAM-A, JAM-B, ICAM-2 and vitronectin, following on from previous studies suggesting that IE adhesion may be supported by a wide array of endothelial receptors^40^. The relative frequency of parasite adhesion to the different receptors varies, with several receptors supporting binding of only a small proportion of the isolates. An earlier study reported that antibodies against integrin α_v_ chain reduced parasite adhesion to human dermal microvascular endothelial cells, suggesting that integrin αvβ3 plays a role in parasite adhesion^12^. Here we directly showed that fresh parasite isolates bind to recombinant integrin αvβ3, and this receptor together with CD36 are the most common endothelial receptors mediating adhesion of fresh isolates (Table 2). A previous study reported that integrin α5β1 alone did not support IE adhesion to endothelial cells, but α5β1 in complex with CD36 increased IE adhesion to endothelial cells under flow conditions^14^. In the current study, we find that IEs from clinical isolates can directly adhere to integrin α5β1 under static conditions, and the different observations could be explained by different assay conditions. Interestingly, hemoglobin HbAC significantly reduced and age significantly increased IE adhesion to both CD36 and integrin α5β1.

IE adhesion under flow was described as a multistep process that includes tethering and rolling followed by firm adhesion, for example rolling on the receptors P-selectin or ICAM-1 and then firm adhesion to CD36 (reviewed in ref.^41^). Some of the novel receptors described here might similarly be part of a multi-receptor adhesion process that involves both rolling and firm adhesion types, rather than unrelated adhesion events. Further, IE binding levels to members of the integrin family, PECAM-1, P-selectin and E-selectin correlated, which could be related to the polyclonality of clinical isolates, multi-receptor binding of individual parasites, or both.

We examined hemoglobin variants for their effects on IE adhesion. While hemoglobin AS has been consistently associated with protection from severe malaria, earlier studies have differed as to whether AS also reduces the risk of infection or the density of parasitemia^17^. The protective effect of hemoglobin AC is less clear, with some studies reporting a non-significant effect on severe malaria risk, and two studies reporting a protective effect against severe malaria or cerebral malaria^17,42,43^. The protective effect of HbAS and HbAC was proposed to be mediated in part by changes in knob structure and PfEMP1 expression by parasitized HbAS and HbAC erythrocytes resulting in a significant reduction in IE adhesion^44,45^. Laboratory isolates cultured in HbAS erythrocytes adhere at a lower level to dermal HMVECs (which is mediated by CD36 and ICAM-1), and to purified CD36 and ICAM-1, although in one study adhesion to HUVECs (which is mediated by ICAM-1 alone) was unchanged^45–47^.

In the current study, hemoglobin AC significantly reduced the level of IE binding to endothelial receptors CD36 and integrin α5β1, while sickle cell trait did not significantly reduce the level of IE binding to any of the endothelial receptors. In this cohort of children, parasite densities were significantly lower among children with hemoglobin AS (p < 0.0001). These results suggest that HbAS confers protection from severe malaria and reduces parasite densities by other mechanisms such as enhanced phagocytosis of ring-stage parasites^48^, while HbAC might confer protection from severe malaria by reducing parasite adhesion.

Blood groups A, AB and B were associated with a significant reduction of IE binding to the receptor ICAM-1 compared to blood group O. The protective effect of blood group O on severe malaria has been inconsistent in earlier studies, although recent genome-wide association studies observed that blood group O reduced the risk of severe malaria^32–37^. We found that IE from children with blood group O bind at significantly higher levels to ICAM-1, an unexpected pattern for a host factor associated with protection from disease. These findings could be explained if the binding interaction involved is unrelated to severe malaria, and/or that the protective effect of blood group O is not mediated by cytoadhesion to endothelial receptors but by other adhesion mechanisms like decreased IE rosetting^38,39^.

A number of receptors have been proposed to mediate severe malaria IE binding^15,22,23^, but no consistent pattern has emerged between studies. Severe malaria was related to ICAM-1-binding parasites in some but not other studies^15,19–22^, and ICAM-1 polymorphisms did not correlate with severe malaria risk^49^. Clinical isolates from children with severe malaria bind EPCR (and ICAM-1) in Tanzania at higher levels^15^, and in Mozambique a higher proportion of IEs from children presenting with seizures bound to the receptor gC1qR/HABP1/p32^23^. In the current study, severe malaria IE adhered at similar levels to all the receptors.

We propose two explanations for the lack of a clear distinction in binding levels to the different receptors between IEs from children with severe versus non-severe malaria. First, earlier studies demonstrated that IEs from children with severe malaria as well as IEs from young children in general are more commonly recognized by plasma from the community, suggesting that these IEs may surface-display a shared repertoire of PfEMP1^50–52^. An overlap in the PfEMP1 repertoire among young children including those with severe malaria could yield similar IE adhesion properties, such as we have observed. Furthermore, age should be assessed for its confounding effects in studies to associate parasite adhesion to malaria severity. Of note, median age did not differ between children with or without severe malaria in the current study, and the rates of severe malaria remained stable post-infancy, such that age should not be a confounding factor in the results reported here.

Second, peripheral blood isolates are likely to be a mixed pool of parasites, which might variably derive from those causing and those not causing a specific disease presentation. For example, in our studies of pregnancy malaria, we showed that placental parasites have a unique binding phenotype and uniformly bind to the receptor CSA. However, peripheral blood parasites from the same women sometimes bind to CD36 as well as CSA, or to CD36 alone, demonstrating that peripheral samples do not always reflect the phenotype of the sequestered parasites causing a specific syndrome. Notably, parasites selected to bind CSA lose the ability to bind CD36, and hence the peripheral blood samples that yield evidence of binding to both CD36 and CSA, are likely to represent mixed pools of parasites, rather than a single pool of parasites that bind multiple receptors.

In summary, the current study has identified several novel receptors for IE adhesion such as JAM-B, cellular fibronectin and laminin, and described distinct binding interactions that are modified by red cell variants, blood group and host age. This large scale study demonstrates the complexity of associating IE binding phenotypes with disease severity. The lack of a significant association between severe malaria and adhesion to any of the receptors suggests that either IE associated with severe malaria do not have a distinct adhesion phenotype compared to IE from young children without severe malaria, or severe malaria IE adhere to a discrete endothelial receptor not included in the panel evaluated in this study.

## Methods

### Study population and clinical definitions

Children participating in this study were enrolled between August 2010 and December 2014 into two longitudinal cohorts conducted as part of the Immuno-Epidemiology (IMEP) project in Ouelessebougou, Mali: 1. A birth cohort of pregnant women and their children; 2. Children aged 0–3 years. A parent or guardian gave informed consent for their child’s participation in the study, after receiving a study explanation form and an oral explanation from the study clinicians in their native language. The protocol and study procedures were approved by the institutional review board of the National Institute of Allergy and Infectious Diseases at the National Institutes of Health (ClinicalTrials.gov ID NCT01168271), and the Ethics Committee of the Faculty of Medicine, Pharmacy and Dentistry at the University of Bamako, Mali. All study methods were performed in accordance with the relevant guidelines and regulations of the institutional review boards.

Active follow-up included clinical examination and blood smear microscopy for detecting malaria parasites, monthly during the rainy season (July-December), every two months during the dry season (January-June), and at any time the child was sick, up to the age of 5 years.

Severe malaria was defined as parasitemia together with at least one of the following WHO criteria for severe malaria: >2 convulsions in the past 24 hr; prostration (inability to sit unaided or in younger infants inability to move/feed); hemoglobin <5 g/dl; respiratory distress (hyperventilation with deep breathing, intercostal recessions and/or irregular breathing); coma (Blantyre score  <=2).

### Adhesion assays

Blood samples were collected every 3 months during the first 2 years of life, every 6 months thereafter, and during every symptomatic malaria infection diagnosed at an unscheduled visit. Samples from malaria-infected children (defined by blood smear microscopy) were used in adhesion assays. Ring stage parasites in blood samples collected from children were allowed to mature to the trophozoite/schizont stages in *in vitro* culture for 16–20 hr. IE were enriched using the gelatin flotation method and adjusted to 0.2–20% parasitemia before binding assay. IE binding to specific receptors immobilized on Petri dishes was determined in a static binding assay. The panel of purified receptors included the following human endothelial molecules: CD36, ICAM-1, VCAM, PECAM-1, E-selectin, P-selectin, integrin α3β1, integrin α5β1, integrin αvβ3, JAM-A, JAM-B, VE-cadherin, vitronectin, ICAM-2 (R&D); TSP, CSA, cellular fibronectin, and laminin from placenta (Sigma, St. Louis, MO); EPCR (My BioSource); EPCR produced in eukaryotic cells^53^ that was provided by Dr. Benoît Gamain. Bovine serum albumin (BSA) (Sigma, St. Louis, MO) was used as a negative control. 20 μl of recombinant proteins (10 μg/ml) were applied to a petri dish (Falcon 351029), and incubated overnight at 4 °C in a humid chamber. Prior to the adhesion assay, receptor solutions were removed and 20 μl of blocking solution (3% BSA in PBS) added and incubated for 30–60 minutes at room temperature. 20 μl of parasite suspension were applied, and after 30 minutes at room temperature, unbound IE and erythrocytes were removed by 3 washes with PBS. Bound IE were fixed with 0.5% glutaradehyde in PBS for 10 minutes and stained with 1% Giemsa for 2 minutes. Bound erythrocytes and IE were counted under 1000x magnification, and 20 fields with the highest IE counts were recorded. Raw binding data is included in Supplementary Table 1.

### Erythrocyte polymorphisms

Hemoglobin types (HbAA, HbAS, HbSS, HbAC, HbCC, HbSC) were determined using Titan III® Cellulose Acetate Plate according to the manufacturer’s instructions (Helena laboratories, Beaumont, Texas, USA).

### Statistical analyses

Data were collected in standardized forms, entered and verified using DataFax (version 4.2, Clinical DataFax Systems, Inc., Hamilton, Ontario, Canada).

The level of IE binding was adjusted for the background by subtracting RBC (E) count for the specific receptor and IE count for BSA (adjusted IE binding = (IE_specific receptor_ − E_specific receptor_ − IE_BSA_), and negative values were converted to 0. The relationships between adhesion to endothelial receptors and severe malaria, age, hemoglobin type, and blood group, were assessed using multivariate linear regression models adjusted for parasite density in the assay. P values of 0.0026 were considered significant to account for the comparison of 19 receptors. The analyses were carried out in R (version 3.3.2).

Incidence rates of severe malaria by age category were compared by Poisson regression model via GEE to account for correlation due to repeated measures for each child. At the individual level, number of severe malaria events were regressed on indicator variables of the age strata along with a log-offset of time at risk in that strata.

The analyses included binding data from freshly collected parasites to relate to WHO defined severe malaria, hemoglobin type, blood group and age for study participants. All data analyzed during this study are included in the article (Supplementary Table 1).

## Electronic supplementary material

Supplementary Information

## References

[1] Riganti M (1990). Human cerebral malaria in Thailand: a clinico-pathological correlation. Immunol Lett.

[2] Fried M, Duffy PE (1996). Adherence of Plasmodium falciparum to chondroitin sulfate A in the human placenta. Science.

[3] Roberts DD (1985). Thrombospondin binds falciparum malaria parasitized erythrocytes and may mediate cytoadherence. Nature.

[4] Barnwell JW (1989). A human 88-kD membrane glycoprotein (CD36) functions *in vitro* as a receptor for a cytoadherence ligand on Plasmodium falciparum-infected erythrocytes. J Clin Invest.

[5] Oquendo P, Hundt E, Lawler J, Seed B (1989). CD36 directly mediates cytoadherence of Plasmodium falciparum parasitized erythrocytes. Cell.

[6] Ockenhouse CF, Tandon NN, Magowan C, Jamieson GA, Chulay JD (1989). Identification of a platelet membrane glycoprotein as a falciparum malaria sequestration receptor. Science.

[7] Berendt AR, Simmons DL, Tansey J, Newbold CI, Marsh K (1989). Intercellular adhesion molecule-1 is an endothelial cell adhesion receptor for Plasmodium falciparum. Nature.

[8] Ockenhouse CF, Betageri R, Springer TA, Staunton DE (1992). Plasmodium falciparum-infected erythrocytes bind ICAM-1 at a site distinct from LFA-1, Mac-1, and human rhinovirus. Cell.

[9] Robert C (1995). Chondroitin-4-sulphate (proteoglycan), a receptor for Plasmodium falciparum-infected erythrocyte adherence on brain microvascular endothelial cells. Res Immunol.

[10] Rogerson SJ, Chaiyaroj SC, Ng K, Reeder JC, Brown GV (1995). Chondroitin sulfate A is a cell surface receptor for Plasmodium falciparum-infected erythrocytes. J Exp Med.

[11] Treutiger CJ, Heddini A, Fernandez V, Muller WA, Wahlgren M (1997). PECAM-1/CD31, an endothelial receptor for binding Plasmodium falciparum-infected erythrocytes. Nat Med.

[12] Siano JP, Grady KK, Millet P, Wick TM (1998). Short report: Plasmodium falciparum: cytoadherence to alpha(v)beta3 on human microvascular endothelial cells. Am J Trop Med Hyg.

[13] Biswas AK (2007). Plasmodium falciparum uses gC1qR/HABP1/p32 as a receptor to bind to vascular endothelium and for platelet-mediated clumping. PLoS Pathog.

[14] Davis SP (2013). CD36 recruits alpha(5)beta(1) integrin to promote cytoadherence of P. falciparum-infected erythrocytes. PLoS Pathog.

[15] Turner, L. *et al*. Severe malaria is associated with parasite binding to endothelial protein C receptor. *Nature* (2013). 10.1038/nature12216 PMC3870021 23739325

[16] Smith JD (2014). The role of PfEMP1 adhesion domain classification in Plasmodium falciparum pathogenesis research. Mol Biochem Parasitol.

[17] Taylor SM, Parobek CM, Fairhurst RM (2012). Haemoglobinopathies and the clinical epidemiology of malaria: a systematic review and meta-analysis. Lancet Infect Dis.

[18] Ockenhouse CF (1991). Molecular basis of sequestration in severe and uncomplicated Plasmodium falciparum malaria: differential adhesion of infected erythrocytes to CD36 and ICAM-1. J Infect Dis.

[19] Ho M (1991). Clinical correlates of *in vitro* Plasmodium falciparum cytoadherence. Infect Immun.

[20] Newbold C (1997). Receptor-specific adhesion and clinical disease in Plasmodium falciparum. Am J Trop Med Hyg.

[21] Rogerson SJ (1999). Cytoadherence characteristics of Plasmodium falciparum-infected erythrocytes from Malawian children with severe and uncomplicated malaria. Am J Trop Med Hyg.

[22] Ochola LB (2011). Specific receptor usage in Plasmodium falciparum cytoadherence is associated with disease outcome. PLoS One.

[23] Mayor A (2011). Association of severe malaria outcomes with platelet-mediated clumping and adhesion to a novel host receptor. PLoS One.

[24] Heddini A (2001). Fresh isolates from children with severe Plasmodium falciparum malaria bind to multiple receptors. Infect Immun.

[25] Goncalves BP (2014). Parasite burden and severity of malaria in Tanzanian children. N Engl J Med.

[26] Wenisch C (1994). Serum laminin in malaria. J Clin Pathol.

[27] Burgmann H (1996). Serum laminin and basic fibroblast growth factor concentrations in patients with complicated Plasmodium falciparum malaria. J Clin Immunol.

[28] Eda, S. & Sherman, I. W. Plasmodium falciparum-infected erythrocytes bind to the RGD motif of fibronectin via the band 3-related adhesin. *Exp Parasitol* **107** (2004). 10.1016/j.exppara.2004.06.002 15363941

[29] Weber C, Fraemohs L, Dejana E (2007). The role of junctional adhesion molecules in vascular inflammation. Nat Rev Immunol.

[30] Navratil E, Couvelard A, Rey A, Henin D, Scoazec JY (1997). Expression of cell adhesion molecules by microvascular endothelial cells in the cortical and subcortical regions of the normal human brain: an immunohistochemical analysis. Neuropathol Appl Neurobiol.

[31] Rask TS, Hansen DA, Theander TG, Gorm Pedersen A, Lavstsen T (2010). Plasmodium falciparum erythrocyte membrane protein 1 diversity in seven genomes–divide and conquer. PLoS Comput Biol.

[32] Martin SK (1979). Frequency of blood group antigens in Nigerian children with falciparum malaria. Trans R Soc Trop Med Hyg.

[33] Montoya F, Restrepo M, Montoya AE, Rojas W (1994). Blood groups and malaria. Revista do Instituto de Medicina Tropical de Sao Paulo.

[34] Fry AE (2008). Common variation in the ABO glycosyltransferase is associated with susceptibility to severe Plasmodium falciparum malaria. Human molecular genetics.

[35] Jallow M (2009). Genome-wide and fine-resolution association analysis of malaria in West Africa. Nature genetics.

[36] Timmann C (2012). Genome-wide association study indicates two novel resistance loci for severe malaria. Nature.

[37] Toure O (2012). Candidate polymorphisms and severe malaria in a Malian population. PLoS One.

[38] Rowe JA (2007). Blood group O protects against severe Plasmodium falciparum malaria through the mechanism of reduced rosetting. Proc Natl Acad Sci USA.

[39] Vigan-Womas I (2012). Structural basis for the ABO blood-group dependence of Plasmodium falciparum rosetting. PLoS Pathog.

[40] Esser C (2014). Evidence of promiscuous endothelial binding by Plasmodium falciparum-infected erythrocytes. Cell Microbiol.

[41] Ho M, White NJ (1999). Molecular mechanisms of cytoadherence in malaria. Am J Physiol.

[42] May J (2007). Hemoglobin variants and disease manifestations in severe falciparum malaria. JAMA.

[43] Modiano D (2001). Haemoglobin C protects against clinical Plasmodium falciparum malaria. Nature.

[44] Fairhurst RM, Bess CD, Krause MA (2012). Abnormal PfEMP1/knob display on Plasmodium falciparum-infected erythrocytes containing hemoglobin variants: fresh insights into malaria pathogenesis and protection. Microbes Infect.

[45] Opi DH (2014). Mechanistic Studies of the Negative Epistatic Malaria-protective Interaction Between Sickle Cell Trait and alpha + thalassemia. EBioMedicine.

[46] Cholera R (2008). Impaired cytoadherence of Plasmodium falciparum-infected erythrocytes containing sickle hemoglobin. Proc Natl Acad Sci USA.

[47] Rowland PG, Nash GB, Cooke BM, Stuart J (1993). Comparative study of the adhesion of sickle cells and malarial-parasitized red cells to cultured endothelium. J Lab Clin Med.

[48] Ayi K, Turrini F, Piga A, Arese P (2004). Enhanced phagocytosis of ring-parasitized mutant erythrocytes: a common mechanism that may explain protection against falciparum malaria in sickle trait and beta-thalassemia trait. Blood.

[49] Fry AE (2008). Variation in the ICAM1 gene is not associated with severe malaria phenotypes. Genes and immunity.

[50] Bull PC (2000). Plasmodium falciparum-infected erythrocytes: agglutination by diverse Kenyan plasma is associated with severe disease and young host age. J Infect Dis.

[51] Nielsen MA (2002). Plasmodium falciparum variant surface antigen expression varies between isolates causing severe and nonsevere malaria and is modified by acquired immunity. J Immunol.

[52] Abdi AI (2016). Global selection of Plasmodium falciparum virulence antigen expression by host antibodies. Sci Rep.

[53] Nunes-Silva S (2015). Beninese children with cerebral malaria do not develop humoral immunity against the IT4-VAR19-DC8 PfEMP1 variant linked to EPCR and brain endothelial binding. Malar J.

